# CogChamps – a model of implementing evidence-based care in hospitals: study protocol

**DOI:** 10.1186/s12913-017-2136-0

**Published:** 2017-03-14

**Authors:** Catherine Travers, Frederick Graham, Amanda Henderson, Elizabeth Beattie

**Affiliations:** 1 School of Nursing, Queensland University of Technology, Victoria Park Road, Kelvin Grove, QLD 4059 Australia; 2 Princess Alexandra Hospital, Queensland Department of Health, 199 Ipswich Rd, Wooloongabba, QLD 4102 Australia; 3 Nursing Practice Development Unit, Princess Alexandra Hospital, Queensland Department of Health, 199 Ipswich Rd, Wooloongabba, QLD 4102 Australia; 4 Dementia Collaborative Research Centre (DCRC): Carers and Consumers, School of Nursing, Queensland University of Technology (QUT), Level 6, N Block, Victoria Park Rd, Kelvin Grove, QLD 4059 Australia

**Keywords:** Dementia, Delirium, Hospitals, Education, Nursing

## Abstract

**Background:**

Delirium and dementia (cognitive impairment; CI), are common in older hospital patients, and both are associated with serious adverse outcomes. Despite delirium often being preventable, it is frequently not recognized in hospital settings, which may be because hospital nurses have not received adequate education or training in recognizing or caring for those with CI. However, the most effective way of increasing nurses’ awareness about delirium and dementia, and initiating regular patient screening and monitoring to guide best practices for these patients in hospital settings is not known. Hence this current project, conducted in 2015–2017, aims to redress this situation by implementing a multi-component non-pharmacological evidence-based intervention for patients with CI, through educating and mentoring hospital nurses to change their practice.

**Methods:**

The development of the practice change component is informed by recent findings from implementation science that focuses on facilitation as the active ingredient in knowledge uptake and utilization. This component focuses on educating and empowering experienced nurses to become Cognition Champions (CogChamps) across six wards in a large Australian tertiary referral hospital. The CogChamps will, in turn, educate other nursing team members to more effectively care for patients with CI. The hospital leadership team are supportive of the project and are directly involved in selecting the CogChamps. CogChamps will be provided with comprehensive education in evidence-based delirium assessment, prevention and management, and practice change management skills. They will receive continuing support from research and education staff about raising awareness, upskilling other staff in delirium assessment and in the adoption of best practices for preventing and managing delirium. Both qualitative and quantitative data are being collected at multiple time-points to evaluate process, impact and outcome, and to provide clarity regarding the most effective aspects of the intervention.

**Discussion:**

This paper describes the study protocol for the implementation of multi-component evidence-based non-pharmacological practices designed to improve the care of older hospital patients with CI. Findings will inform subsequent initiatives directed towards enhancing the capacity of the nursing workforce to implement best practices for providing high quality care for this growing patient population throughout their acute care hospital stay.

## Background

Cognitive impairment (CI) including dementia and delirium are common amongst older people admitted to acute hospitals [1–3]. Patients with these conditions have a significantly increased risk of serious adverse outcomes when hospitalized, compared with same-aged patients without CI. For instance, dementia is a key risk factor for the development of delirium (patients with dementia have a five to six-fold increased risk of developing delirium than other patients of the same age), which is associated with high morbidity and mortality in older people [4]. Other adverse outcomes associated with both dementia and delirium include cognitive and functional decline, medical and surgical complications, significantly increased risk of falls and longer hospital stays and greatly increased hospital costs [2, 4, 5].

Despite their high prevalence and associated adverse outcomes, dementia and delirium are frequently not recognized or recorded in acute hospitals with delirium being missed in as many as 75% of cases [6–9]. Consequently, appropriate prevention and management strategies [6, 8], are not implemented, which effectively prevent delirium in many at-risk patients [4, 10]. An important reason for the under-recognition of delirium is that nurses working in acute hospitals do not receive adequate or appropriate education in identifying and caring for patients with CI [1, 11]. Accordingly, addressing this issue and ensuring that staff are knowledgeable and adequately skilled to identify and care for these patients has been identified as a key strategy for improving these patients’ care [1, 11–15].

Currently, however, the most effective way of educating staff and initiating practice change for older patients with CI in the hospital environment is unclear, as limited research has been undertaken in this area. Of the studies that have been reported, results generally indicate that, while education can improve knowledge of dementia [13, 16], and delirium [17], education in isolation has little effect on clinical practice [18]. By comparison, the use of knowledge translation principles has been shown to be more effective in producing practice change and preventing delirium [10, 18, 19], and are recommended [20, 21].

The protocol for the implementation of multi-component evidence-based non-pharmacological practices by nurses in the acute hospital setting and the evaluation of its impact and effectiveness in achieving practice change are described in this manuscript. It is based on the three constructs of evidence, context and facilitation identified as critical to effective practice change [22]. Findings and lessons learned from this project will address some of the gaps in the existing evidence base for educating and developing the capacity of the nursing workforce to provide high quality care for older patients with CI.

## Methods

A well-designed implementation plan that acknowledges the interplay of a range of factors: namely the context (e.g., the characteristics of the individuals involved at the local level, the unit culture, attitudes about older people, the middle management and leadership style, and the broader organizational context, such as initiatives and policy drivers); the need for adequate resources and support for facilitation processes; and a sound evidence base for the proposed changes is vital for the successful implementation of change in clinical practice [21]. Accordingly, all of these elements were addressed in developing the protocol (i.e., the implementation plan), that includes clearly identified outcomes and a timeline for completion.

### The evidence

The educational component of this intervention and the non-pharmacological interventions are based on evidence based guidelines for the care of patients with CI in the clinical setting [23, 24], and sound evidence that appropriate prevention and management strategies are effective in preventing delirium in many at-risk patients [4, 10].

### Context and leadership engagement

The implementation of multi-component evidence–based non-pharmacological practices is being progressed in a large tertiary referral hospital - the Intervention Hospital (IH), located in in South-East Queensland, Australia where two of the study’s Chief Investigators (AH, FG) work. Implementation is across four medical and two surgical wards.

Adequate resources and support are available for the project via an Australian Government grant that allows project staff to dedicate sufficient time to assist hospital staff implement and evaluate activities. Executive level support for the project has been obtained by the establishment of a Steering Committee at project commencement. Key hospital staff, including the Director of Internal Medicine, the Directors of Nursing (Medicine, Surgery), and the Nurse Managers from each IH ward are all members (all accepted the invitation) of the Steering Committee. A consumer representative has also been invited to become a member of the Committee via the Consumer Directed Research Network. The purpose of the Committee is to provide support and guidance regarding the project’s implementation. Where appropriate, Steering Committee members may also be asked to assist with managing organizational processes to facilitate project activities (e.g., rostering of CogChamps to attend workshops, and the inclusion of CI project updates in regular ward meeting agendas).

Importantly, the timing of this implementation coincides with the adoption of dementia as a National Health Priority by key Australian healthcare organizations [25] and the designation of improving the care of older patients with CI (dementia and delirium) in acute hospitals as a priority issue. To this end, the Australian Commission on Safety and Quality in Healthcare (ACSQHC), launched a Caring for Cognitive Impairment Campaign in 2016 to guide improvements [26]. As part of the campaign, the ACSQHC has developed a range of sound evidence-based resources that hospitals and clinicians may use to guide their improvement efforts. The ACSQHC also asks that hospitals publicly commit to the campaign, and the names of the hospitals that commit are published on the Cognitive Care Campaign’s website. Importantly, the IH has committed to the campaign. In addition, the ASQHC is currently revising Australia’s National Standards for healthcare organizations to include screening for CI, and the implementation of strategies to prevent delirium and manage CI as mandatory. It is anticipated that the revised standards will be released within the next three years and healthcare organizations will be required to demonstrate their compliance with the Standards in order to maintain their accreditation. Hence, there is an important and timely incentive for the IH executive to support the project.

### Readiness of clinical areas

The current implementation builds upon a number of initiatives previously introduced in the IH to improve the quality of care for patients with CI when hospitalized. This includes the employment of a Clinical Nurse Consultant – Dementia and Delirium who, for the past seven years, has provided an education and consultation service for nurses regarding the care of patients with CI. Over that time, approximately 800 of the IH’s nurses (approximately 25% of the total nursing workforce) have received education (around 7 h) about dementia, delirium and caring for these patients. Of those, 110 expressed an interest in becoming Cognition Champions to promote best practice in caring for patients with CI (a precursor to the current project). The self-determined role of the champions is to assist other staff with strategies for managing hospitalized patients with CI and to develop and promote the adoption of resources which may support care delivery.

Other initiatives include the development of ‘Cognition Corners’ on most wards and the use of a Sunflower chart to aid in person-centered communication with people identified as having CI. Each Cognition Corner houses a range of recreational resources specifically for use by patients with CI (e.g., cards, books, puzzles, soft toys), to manage boredom or distress. The Sunflower is placed (with the patient or carer’s consent) on the wall above the patient’s bed and displays important biographical information about the person including their preferred name, places they have lived, their interests, previous occupation and other important ‘life-story’ information. The chart facilitates person-centered care by providing readily available topics for conversation that are meaningful to the patient. Although anecdotal evidence indicates the initiative has resulted in some positive outcomes (e.g., improved staff morale), it has not been formally evaluated, and has been subject to attrition. Thus, the current CogChamps project is being implemented in a setting in which the care of patients with CI has already been identified as an important issue, hence establishing a solid foundation for this project.

### Processes to facilitate changed practices to promote high quality care for patients with CI

#### Recruiting CogChamps

CogChamps are nurses recruited to champion best practice care for older patients with CI (dementia and delirium) in hospital, and a new cohort has been identified for this project. In the first instance, experienced nursing staff from each of the six IH wards who were willing to become Cognition Champions (CogChamps), were identified. CogChamps were required to have over two years clinical experience and (a) a specific interest in dementia and delirium, or (b) have leadership skills. The identification process was a collaborative venture between the nurse unit managers and the research team. CogChamps will be provided with comprehensive dementia and delirium education and training (Workshop 1) and education about necessary leadership and change management skills (Workshop 2), to facilitate changing care practices.

### Educating CogChamps

Workshop 1 topics include:

- ➢ Project Overview;
- ➢ Dementia versus delirium,
- ➢ Important issues associated with CI in hospital,
- ➢ Behavioral and psychological symptoms of dementia,
- ➢ The assessment of delirium using the Confusion Assessment Method (CAM) [27], This 2 h session will include a demonstration of the CAM by the presenter (FG), practice using the CAM by the CogChamps in pairs, as well as supervised real-life practice by each CogChamp on their home ward. Supervision will be provided by either a hospital Geriatrician or a nurse well versed in use of the CAM, who will observe each CogChamp administer the CAM and interpret the results (with discussion and feedback);
- ➢ Delirium prevention and management;
- ➢ Pharmaceuticals;
- ➢ Implementing change in the workplace: Participants will be introduced to the modified Knowledge Translation (KT) framework [28]. The model outlines four steps for effective knowledge translation: awareness, agreement, adoption, and adherence. Participants will be asked to complete a homework activity that will involve considering which KT stage both they and their ward are at currently, and outline the steps required to transition from that stage to the next stage in the continuum. The activity will be completed prior to, and in preparation for, Workshop 2.

Workshop 2 topics include:

- ➢ What is a Cognition Champion?
- ➢ Elements of change and how to influence it,
- ➢ Development of a ward specific Action Plan by the CogChamps (2 h session),
- ➢ Communication skills including assertive communication, setting clear expectations, and tools and strategies for providing feedback.

A key component of Workshop 2 will be the development of ward specific Action Plans by the CogChamps. CogChamps will be asked to identify areas in their home wards where the care of patients with CI could be improved, and develop specific plans to make those improvements. This will ensure that the specific interventions to be adopted address the local needs of each ward and the preferences of the CogChamps working on those wards, thereby promoting engagement in the process and ownership of the Action Plan [21] (pps 43,88) Members of the research team will subsequently meet with the CogChamps from each ward to refine the Action Plans and identify three specific, actionable items/steps with timelines for implementation and the identification of the CogChamp(s) responsible. Three action items are considered achievable within the confines of each ward’s resources and within the project’s timeframe (4–6 months for the implementation of Action Plans). CogChamps will also be encouraged to include success measures in their plans, for example, if educating other nurses on the wards in correctly using the CAM is an action item on their plan, they will be encouraged to collect data regarding the number of nurses who receive such education as well as pre- and post knowledge data (Research staff will assist with the compilation of data and feedback of results).

Both workshops will be full-day workshops (7 h), and employ sound educational strategies to actively promote learning and understanding, namely factual information, interactive workshops, role plays, and case study discussion [13, 29]. All sessions will be delivered by an expert in the relevant field (e.g., the Hospital Pharmacist will present the pharmacy component, i.e., medication use in older patients with CI), and participants will receive continuing practice development points and a certificate for participation. Workshop One focuses on providing participants with the knowledge and skills to care for patients with CI, bearing in mind they have some prior knowledge of dementia and delirium. By comparison, Workshop Two is based on an established preceptorship workshop regularly provided to experienced nurses to further develop their leadership skills at the IH, but tailored to address the specific objectives of this project.

### Enabling CogChamps

The CogChamps research team will support the CogChamps throughout the implementation by providing resources (e.g., educational materials, questionnaires) and the appointment of facilitators to support them in their roles. Facilitation involves assessing, aligning and integrating evidence with opportunities in the clinical setting and facilitators support staff in the care setting to adopt innovations by assisting them to tailor the innovation to the particular setting [30]. There is good evidence that facilitators increase the likelihood that practice change will be adopted, when they are supernumerary and sufficiently prepared for their role [31].

Three facilitators will be employed for this project including one external experienced (i.e., expert) and two internal ‘novice’ facilitators. The expert facilitator has both clinical nursing experience together with knowledge and experience of adopting quality improvement projects in hospital settings. Their role will be to assist CogChamps to maintain focus on the project and support them to implement their ward specific Action Plans. Specifically, they will meet briefly (10–15 min) with the CogChamps on a regular basis (ideally weekly, but at least fortnightly) to:

- ➢ Mentor CogChamps to implement their Action Plans,
- ➢ Ascertain and document progress towards the implementation of each ward’s Action plan,
- ➢ Identify any barriers to progress, and guide CogChamps to identify possible solutions,
- ➢ Provide feedback to CogChamps regarding their progress, and
- ➢ Assist CogChamps to develop processes essential to effective project implementation (e.g., systems for communicating between CogChamps on each ward who work differing rosters).

Two of the newly recruited CogChamps (one from each of the medical and surgical wards), will also be seconded to work full-time on the project as internal ‘novice’ facilitators. They will work in this capacity for four to six weeks and while they are inexperienced facilitators, they have the advantage of having intimate knowledge of the hospital setting and the staff involved. They will be mentored in their new role by a member of the research team (AH) and will undertake a very active role in assisting and mentoring CogChamps to progress their action plans, including assisting nursing staff to complete CI assessments at the bedside and guide them in developing a plan of care based on best practices.

In addition to the appointment of the external facilitator, the research team will maintain a high level of visibility on the wards throughout the project’s implementation by regularly visiting the wards (approximately weekly) and via email contact (e.g., to inform CogChamps about resources, etc.) to reinforce suggestions/decisions and assist overcome barriers.

Data (process and outcome) will be collected on multiple occasions throughout the practice implementation to evaluate its impact and effectiveness, and to identify key component(s) for effectiveness.

### Sustainability

Strategies to ensure the longer-term sustainability of the project were considered from the outset. As the embedding of new processes and procedures in everyday activities to routinize them is essential for achieving sustainability [21] (p44) the CogChamps will be encouraged to consider how practice changes might be embedded within their everyday routines (e.g., CI to be included as a vital sign that is routinely discussed at patient hand-over). They will also be encouraged to develop plans for continuing CogChamps activities following completion of the evaluation component of the project.

Key project activities and timeline are summarized in Table 1.

**Table 1:** CogChamps – Project Timeline and Key Activities

Timeline	Activity
12 months prior to project commencement	Preparation of funding application. The project plan and timeline were prepared well in advance of project commencement. The hospital executive has agreed to support the project should the funding application be successful.
6 months prior to project commencement	Build buy-in. Two members of the research team who work at the IH (FG, AH) liaised with the Nurse Unit Managers of each ward, and Nursing Directors to inform them of the project and garner their support.
At project commencement	Establishment of a Steering Committee. To provide support, guidance and expertise for the project at the Executive level. The Steering Committee will meet regularly throughout the project and will be provided with regular project updates via email.
At project commencement	Nurse Unit Managers were asked to nominate experienced nurses with an interest in cognitive impairment or nurses with leadership potential, to become Cognition Champions (CogChamps). Six nurses from each intervention ward (including some Nurse Educators) have been identified and have agreed to become CogChamps
Pre-intervention	Data Collection 1 (baseline) – pre-intervention Details of the data collected and instruments used are provided in Table 2.
Intervention(Education) – Educating and empowering the CogChamps	The CogChamps will participate in two full day workshops, which will be held approximately 2 months apart (due to rostering issues). Workshop 1 will focus on dementia and delirium education. Workshop 2 will focus on Preceptorship training and the development of ward specific Action Plans by the CogChamps. In the month following workshop 1, each CogChamp’s competency in administering and interpreting the CAM will be consolidated by having an expert conduct a second live observation of the CogChamp performing a CAM. If necessary, the observation will be repeated until the CogChamp is deemed to be competent in administering and interpreting the CAM.
Post Education Intervention	Data Collection 2 – Post CogChamps Workshops
Intervention (Implementation)– Implementation of Action plans by CogChamps 4–6 months	CogChamps will be supported to (1) refine the Action plans developed in Workshop 2, and (2) implement their Action Plans. Appointment of facilitators to support and mentor the CogChamps. The external (expert) facilitator will maintain regular (at least fortnightly) contact with the CogChamps. Two CogChamps will be seconded from their home wards (one from each of the medical and surgical wards) to work full-time on the CogChamps project for a period of 4–6 weeks.
Post – Implementation	Data Collection 3 – Post Intervention Withdrawal of the Research Team from CogChamp activities Qualitative interviews with CogChamps
3 months Post Implementation Intervention	Data Collection 5 - Follow-Up data collection

### Evaluation of implementation activities

The impact and effectiveness of the intervention at the IH will be assessed by comparing the uptake of best practices at this site with another comparable site, located nearby (approximately 10 km away). Two wards (one medical, one surgical) in this hospital will serve as control wards (Control Hospital; CH), and the two hospitals are comparable insofar as nursing staff have been exposed to the same educational modules for dementia and delirium as IH nurses and they have similar patient profiles.

The same data will be collected at both sites.

### Expected outcomes and project evaluation

Expected project outcomes include:

- ➢ Increased nurses’ knowledge of CI and self-confidence in nursing patients with CI;
- ➢ Increased number of nurses in the IH who are proficient in assessing and documenting dementia and delirium;
- ➢ Increased number of patients assessed for CI at admission to hospital (IH);
- ➢ Improved outcomes of nursing care practices for older patients with CI (e.g., improved pain management, nutrition & hydration, patient mobilization) [12, 24], and
- ➢ Reduced adverse outcomes for older patient with CI in hospital (i.e., falls, antipsychotic use).

Each of these outcomes is being evaluated as outlined in Table 2.

**Table 2:** CogChamps: Expected project outcomes and evaluation methods

Expected outcome	Evaluation Method	Tool/Details
Increased Nurses’ knowledge of dementia and delirium	Delirium knowledge questionnaire administered immediately prior to Workshop 1, and re-administered immediately prior to Workshop 2. CogChamps will also be encouraged to administer this tool to other nurses on their wards prior to, and following any CI education they undertake, if CI education is included in their ward’s Action Plan.	The questionnaire includes 15 True/False items relating to delirium features and risk factors, and five validated vignettes, developed specifically for nurses [34]. Five vignettes will be included in the baseline questionnaire and another five (matched for diagnostic complexity) will be administered at follow-up. True/False items - As a literature search failed to identify any well validated tools for assessing nurse’s delirium knowledge, the 15 items common to the Delirium Knowledge Questionnaire [35] and an assessment tool developed by Wand and colleagues [36] were selected.
Increased Nurses’ self-confidence when nursing patients with delirium or confusion	Single item assessing nurses’ self-confidence administered immediately prior to Workshop 1 and re-administered immediately prior to Workshop 2.	Single item statement answered using a 1–5 scale where 1 = not at all confident and 5 = very confident. The item was a slight adaptation of a previously used item [37].
Increased number of nurses at the IH who are proficient in assessing delirium.	Direct observation of CAM administration and interpretation by an expert. Following Workshop 1.	Proficiency will be established by observing CogChamps administer a CAM to a patient and interpret it.
Increased proportion of older patients who are routinely assessed for CI at admission to the hospital.	Room & Chart Audit/Observation tool These data will be collected on multiple occasions throughout the project – (1) Pre- intervention, (2) Following the CogChamps training, (3) Following the implementation of ward specific Action Plans, and (4) Three months following withdrawal of the research team.	The room & chart audit/observation tool included an item regarding cognitive assessment – ‘There is documentation that the patient’s cognitive function was assessed using a standardized assessment tool within 24 h of admission to the ward’. This item was adapted from a similar item developed by Schnitker and colleagues for use in the hospital Emergency Department [38].
Increased implementation of best practice guideline for delirium prevention, management and treatment.	Audits of patient rooms and charts. These data will be collected on the same four occasions as the previous item. Direct observations of Nurse: patient interactions.	The room & chart audit/observation tool includes questions relating to cognitive assessment, pain assessment and management (e.g., Was a pain assessment undertaken? Had analgesia been administered within the last 24 h?), and antipsychotic/benzodiazepine use (Was the patient prescribed or administered any PRN antipsychotic/benzodiazepine medication within the past 24 h?). Items requiring direct observation include aspects of the environment (e.g., Was there a clock set to the correct time, that the patient could see from his/her bed?); nutrition (Was adequate assistance provided to the patient if the patient had difficulty eating or drinking); restraint use (Was the patient restrained?); use of indwelling catheters (IDC; Did the patient have an IDC in situ?), communication (If the patient exhibited confusion/dis-orientation, did the nurse say anything to re-orient the patient?), and patient activity (What was the patient doing when you entered the room?).
Older patients with CI will have fewer adverse outcomes.	Data regarding adverse outcomes from the hospital’s administrative database will be extracted at each data collection point and compared across data collection points and between the IH and CH.	Data regarding falls and antipsychotic use will be obtained from the hospital’s database.

### Evaluation tool

#### The room & chart audit/observation tool

The audit/observational tool was designed to capture data regarding best practice nursing care of older patients with CI, particularly care processes relating to delirium prevention and management. The tool was informed by key Australian guidelines and documents [12, 23], and was initially developed by the lead author (CT) and subsequently revised by the research team until consensus regarding the included items was reached. Most items require an objective Yes/No/Not applicable answer.

#### Procedure for collecting evaluative data

The audits/observations will be completed by two Research Nurses who will be seconded from other hospital wards at the IH (and hence will be familiar with the hospital and its processes), to work on the project. They will be trained to use the tool by CT, and practice cases will be completed (not for inclusion in the final dataset) until an inter-rater agreement of 90% is achieved [32]. The same two nurses will collect the data on each occasion.

The audits/observations will be undertaken for one full day in each of the six intervention and two control wards on each of the five data collection occasions (i.e., a total of 40 days). The data collection is scheduled to occur over the second half of the month on each occasion, and the specific ward to be audited for the day will be randomly selected, using SPSS’s randomization function. One week prior to data collection, each ward’s Nurse Unit Manager will be informed via email, of the day their ward will be scheduled for data collection.

On each audit morning, the Research Manager (CT) will identify all patients with a documented diagnosis of dementia or delirium, or report of confusion, memory problems or other CI in the patient’s chart, or reported verbally by the Charge Nurse. While it is acknowledged that a formal assessment process would be required to be completely confident that all patients with CI are included, this method described is considered adequate for this study. Moreover, the inclusion of patients identified by a nurse with first-hand knowledge of the patients as likely having CI, will overcome some of the under-reporting of dementia in hospital records [8].

A maximum of eight patients will be selected per day, and allocated equally to each Research Nurse. Where more than 8 patients are identified on a particular day, eight patients will be selected using a commercially available random number generator (42 Random Number) APP downloaded onto an Android smart phone. Each patient will be audited/observed on four separate occasions throughout the day: early morning (7 am–9.30 am), late morning (9.30 am–12.00 noon), early afternoon (12.00 noon–1.30 pm) and mid-afternoon (1:30 pm–3:00 pm). The Research Nurse will be required to remain in the patient’s room for at least 15 min per observation and at least one meal-time will be observed. Research Nurses will be instructed to conduct the observations as unobtrusively as possible, and to be polite if patients speak to them but not to encourage conversation.

As no personally identifying information will be recorded for either nurse or patient, obtaining individual informed consent from each participant is not required by the relevant Ethics Committees. Hence, the participation rate is expected to be very high and patients will be excluded only if the patient is likely to be absent from their room for much of the observation period (e.g., a procedure is scheduled).

### Process measures

Process measures that will also be used to evaluate the study’s impact include:

- ➢ The degree to which Action Plans are implemented, including the number of nurses who receive education about CI, and other activities undertaken, and
- ➢ Progress notes maintained by the facilitators.

In addition, qualitative data regarding the project and its impact will be collected via semi-structured interviews with the CogChamps at the conclusion of the project. Their opinions regarding the usefulness of the intervention will be solicited; they will also be asked how much they have learnt about dementia, delirium and caring for patients with CI through their involvement in the project, whether they have observed any changes in nursing practices on their ward and what (if any) impact these changes have had on patients and their behavior. Additionally they will be asked which strategies were most effective for assisting patients with CI, whether there were any barriers to implementing the interventions and suggestions for improving the intervention.

## Discussion

This paper describes, in detail, the rationale and methods of an implementation plan designed to educate and empower nurses to change practice for patients with CI using a multi-component best evidence non-pharmacological intervention. Few dementia and delirium education and training programs for the acute hospital setting have been evaluated and this project’s findings will provide important information regarding the feasibility and effectiveness of our approach of implementing best practices for patient care in this setting. The collection of direct observations of nurse-patient interactions will provide direct evidence of practice change, which, to the best of our knowledge, has not previously been undertaken in studies of this kind. The collection of data at key time-points throughout the project will also provide important insights regarding which elements of the implementation process are most important for achieving any changes observed. Moreover, the data regarding the implementation of activities collected by the facilitators throughout the project will allow us to make inferences regarding the ‘dose’ required to promote practice change.

### Challenges faced

This project faces the usual well-documented challenges associated with the implementation of practice change in the busy hospital environment including heavy workloads, competing demands, the complexity of the care required and the care environment itself, limited buy in from senior leadership, the possibility that nurses may not be committed to the project as well as the challenges associated with shift work and possible attrition. Knowledge of these likely barriers at the project’s outset has allowed us to factor them in at the planning stage and address some pre-emptively. For instance, the potential lack of commitment or ownership in the project is being addressed by having the CogChamps develop and assume responsibility for their own Action Plans, while attrition is being addressed through strategic recruitment throughout the project. The early engagement and commitment of nursing and medical leaders has formed part of the project from its inception.

### Study strengths and weaknesses

An important strength of this study is the collection of multiple sources of data including process measures which will provide rich data regarding the project’s impact including the identification of elements that may account for any observed impacts. Factors likely to contribute to the projects’ success include the adequacy of project funding, and development of a clear project plan together with key targets for change and timelines. Also important is the timing of the project which is occurring at a time when the care of hospitalized patients with CI has been identified as a priority issue – so much so that a national Cognitive Care campaign has been launched, to which the IH has made a public commitment.

One limitation of our intervention is that the physical environment which is known to impact the care of hospitalized patients with CI, is not addressed [33]. However, this is outside the scope of the project, although it is acknowledged that modifying the built environment to make it more ‘dementia-friendly’ is an important component of improving the care of patients with CI when hospitalized.

## Conclusion

This project to educate nurses and develop their capacity to implement best practices for patients with CI when hospitalized represents an ambitious attempt to improve the care of older hospital patients in a complex environment. The results of the evaluation and lessons learned will add to the existing evidence base surrounding practice change and will inform future projects that aim to educate and develop the capacity of the nursing workforce to provide high quality care for older patients with CI from the point of admission through discharge.

## Abbreviations

ACSQHC: Australian Commission on Safety and Quality in Health Care
CH: Control Hospital
CI: Cognitive Impairment
CogChamps: Cognition Champions
IH: Intervention Hospital
KT: Knowledge Translation
